# Development and validation of a nomogram for the risk prediction of malignant cerebral edema after acute large hemispheric infarction involving the anterior circulation

**DOI:** 10.3389/fneur.2023.1221879

**Published:** 2023-09-14

**Authors:** Wei Xie, Xiaoming Ma, Geman Xu, Yumei Wang, Wendie Huang, Meng Liu, Shiying Sheng, Jie Yuan, Jing Wang

**Affiliations:** ^1^Department of Neurology, The Third Affiliated Hospital of Soochow University, The First People's Hospital of Changzhou, Changzhou, China; ^2^North China University of Science and Technology, Tangshan, China; ^3^Department of Neurology, Affiliated Fuyang People's Hospital of Anhui Medical University, Fuyang, China; ^4^Institution of Mental Health, North China University of Science and Technology, Tangshan, China; ^5^Jitang College, North China University of Science and Technology, Tangshan, China; ^6^Clinical Department, Tangshan Vocational and Technical College, Tangshan, China; ^7^Tangshan Union Medical College Hospital, Tangshan, China

**Keywords:** stroke, nomogram, malignant cerebral edema, large hemisphere infarction, ischemic stroke

## Abstract

**Background:**

Malignant cerebral edema (MCE) is a life-threatening complication of large hemisphere infarction (LHI). Therefore, a fast, accurate, and convenient tool for predicting MCE can guide triage services and facilitate shared decision-making. In this study, we aimed to develop and validate a nomogram for the early prediction of MCE risk in acute LHI involving the anterior circulation and to understand the potential mechanism of MCE.

**Methods:**

This retrospective study included 312 consecutive patients with LHI from 1 January 2019 to 28 February 2023. The patients were divided into MCE and non-MCE groups. MCE was defined as an obvious mass effect with ≥5 mm midline shift or basal cistern effacement. Least absolute shrinkage and selection operator (LASSO) and logistic regression were performed to explore the MCE-associated factors, including medical records, laboratory data, computed tomography (CT) scans, and independent clinic risk factors. The independent factors were further incorporated to construct a nomogram for MCE prediction.

**Results:**

Among the 312 patients with LHI, 120 developed MCE. The following eight factors were independently associated with MCE: Glasgow Coma Scale score (*p* = 0.007), baseline National Institutes of Health Stroke Scale score (*p* = 0.006), Alberta Stroke Program Early CT Score (*p* < 0.001), admission monocyte count (*p* = 0.004), white blood cell count (*p* = 0.002), HbA1c level (p < 0.001), history of hypertension (*p* = 0.027), and history of atrial fibrillation (*p* = 0.114). These characteristics were further used to establish a nomogram for predicting prognosis. The nomogram achieved an AUC-ROC of 0.89 (95% CI, 0.82–0.96).

**Conclusion:**

Our nomogram based on LASSO-logistic regression is accurate and useful for the early prediction of MCE after LHI. This model can serve as a precise and practical tool for clinical decision-making in patients with LHI who may require aggressive therapeutic approaches.

## 1. Introduction

Large hemispheric infarction (LHI), which is interchangeably known as malignant middle cerebral artery (MCA) stroke according to its early description in 1996, is a severe form of ischemic stroke affecting most or the entire distribution area of the MCA with or without infarctions in other vascular territories (1). The diverse definitions of LHI have been employed in clinical trials, and they are generally based on a combination of neurological signs or symptoms (2). Furthermore, LHI is associated with a high likelihood of evolving into life-threatening edema, which has been linked with brain herniation. Malignant cerebral edema (MCE) has been reported to develop in >50% of patients with LHI, usually from 1 to several days after the LHI onset (3). Untreated MCE can lead to transtentorial herniation, neurologic deterioration, and death in 40–80% of patients within the 1st week (4).

Cerebral edema develops shortly after the interruption of perfusion and evolves in stages, with a tendency to develop rapidly within 24–36 h and peak at 3–5 days after stroke. Simard et al. were the first to classify cerebral edema according to the following four types: cellular edema, ionic edema, angiogenic edema, and hemorrhage conversion (5). The key mechanisms for MCE development include blood–brain barrier (BBB) breakdown and predominant vasogenic edema formation. Moreover, the degree of cerebral edema is gradually aggravated with increased capillary permeability.

Large hemisphere infarction is characterized by progressive cerebral edema and mass effect, including ipsilateral sulcal effacement, compression of the ipsilateral ventricular system, and a midline shift of structures, such as the septum pellucidum and pineal gland. Additionally, blockage of the foramen of Monro or the third ventricle might occur, resulting in entrapment and dilatation of the contralateral lateral ventricle and obstructive hydrocephalus, which in turn leads to increased intracranial pressure (ICP). However, the complex pathophysiology of LHI development indicates that volume alone should not be considered in MCE prediction. Moreover, some patients with large strokes are more likely to die due to brain herniation than other causes; thus, improved methods are needed to stratify the risk of MCE development.

Although recent advances have been made in ischemic stroke management, treatment options for patients with LHI remain limited. Decompressive hemicraniectomy (DHC) is the only reported successful intervention for relieving the effects of increased ICP and deleterious shifts in the brain tissue (6). Furthermore, DHC has been shown to reduce mortality and improve functional outcomes in several controlled clinical trials; however, an early diagnosis is essential for this procedure. Therefore, identifying predictors of a malignant course in LHI and accurately predicting patient prognosis could provide appropriate and timely treatment and aid decision-making between physicians and families, which is paramount. Predictors of MCE can be broadly categorized into the parameters of neuroimaging, serum markers, and clinical symptoms. These predictors include the initial National Institutes of Health Stroke Scale (NIHSS) score, Alberta Stroke Program Early Computed Tomography Score (ASPECTS), collateral score (CS), clot burden score, diffusion/perfusion parameters on magnetic resonance imaging (MRI), hyperdense artery sign, elevated blood glucose levels, circulating markers that relate to the BBB, leukocytosis, decreased level of consciousness, early nausea and vomiting, female sex, and congestive heart failure.

A nomogram or nomograph is a calculation chart widely used in medicine and comprises scales containing the values of three or more mathematical variables (7). A nomogram can be described as a graphical representation of a clinical prediction model built on multivariate regression analysis. This is conducted by assigning scores based on predictive variable values, calculating the total scores, and converting them to estimate the outcome risk. Such nomograms can predict MCE individually according to each patient's condition. Moreover, traditional models enrolled NIHSS and midline shift (MLS) as categorical variables for convenient clinical use, thereby degrading their precision. However, nomograms can eliminate this limitation. Furthermore, research involving column charts in the field of stroke is currently increasing. Therefore, an intuitive digital interface with improved accuracy and multiple predictive indicators to jointly predict clinical events can help clinical doctors make reasonable and timely decisions and provide explanations that are more intuitive to patient families during doctor–patient communication.

In this study, we applied logistic LASSO regression to identify clinical, laboratory, and radiographic factors that predicted MCE in a broad population of patients with LHI. Furthermore, we employed a nomogram to develop a simple and reliable prediction tool using biomarkers on admission for individual prediction.

## 2. Materials and methods

### 2.1. Study patients

Patients with LHI were continuously enrolled in the Department of Neurology, the Third Affiliated Hospital of Soochow University from 1 January 2019 to 28 February 2023. The study was approved by the Ethics Review Board of the Third Affiliated Hospital of Soochow University (2023-S-080). Additionally, the requirement for informed consent from the study patients was waived due to the retrospective study design.

Patients were included in the study if they met the following inclusion criteria:

1. Diagnosis of acute ischemic stroke.
2. Lesion site included the blood supply area of the MCA with or without additional affected regions, and the cerebral infarction area was at least two-thirds of the blood supply area of the MCA.
3. Age of ≥18 years.
4. Time from onset to admission being <72 h.
5. Vascular recanalization therapy, such as thrombectomy or thrombolysis, was not administered.

Patients who previously experienced severe organ dysfunction or were diagnosed with major medical conditions, including cancer, were excluded from the study.

### 2.2. Data collection

We comprehensively collected information about patient demographic characteristics, clinical variables, vascular risk factors, and laboratory findings. Although a certain amount of data was absent, the ratio of absent information was within an acceptable threshold. Additionally, we executed multiple imputation techniques to fill in the gaps in the MCE and non-MCE groups. Further specifications are provided in Supplementary Table 1.

### 2.3. Definition of MCE

Patients were categorized into the MCE group if they exhibited the following three traits:

1. The presence of neurological dysfunction or a decline in cognitive function and deterioration of consciousness (An increase of ≥1 point in the level of consciousness item [1a] of the NIHSS or an increase of ≥2 points in the overall NIHSS score).
2. A displacement of ≥5 mm in the midline position of the pineal gland/septum pellucidum on CT or MRI imaging.
3. A low-density lesion in the MCA territory accompanied by local signs of brain edema, such as sulcal effacement or lateral ventricle compression.

### 2.4. Statistical analysis

Patients were divided into the MCE and non-MCE groups depending on whether they had developed MCE. The missing dataset values from both groups were subjected to multiple imputations. To investigate the differences between the two groups, categorical variables were represented as counts and composition ratios, whereas continuous variables were presented using means and standard deviations. The inter-group comparison of the categorical variables was conducted with the continuous correction chi-square test, while the continuous variables were compared via one-way ANOVA.

To analyze the full datasets, we implemented LASSO regression to identify and isolate significant components within a complex system of variables. This statistical technique is particularly useful when working with many variables and can help mitigate the collinearity effects between variables. LASSO regression is a type of linear regression that uses a regularization technique (8) for data dimension reduction, feature selection, and radiomics signature building. The LASSO regression model is particularly useful when dealing with high-dimensional and multicollinear datasets. Thus, LASSO regression can efficiently screen for multiple variables using a small sample size, whereas traditional linear regression models cannot handle such datasets. A 10-fold cross-validation test was used to establish the definitive lambda value, and the complete dataset was randomly divided into a training group and a validation group in a 7:3 ratio.

All statistical tests were two-sided, and the significance level was set at a *p*-value of <0.05. All statistical works were conducted on R 4.2.3.

### 2.5. Nomogram development

The variables that exhibited non-zero coefficients in the LASSO regression were employed in the logistic regression on the training set. A backward stepwise regression method was then applied utilizing Akaike information criterion (AIC) evaluation metrics to streamline the number of variables in the logistic regression without compromising model performance. Additionally, the variance inflation factor was used to assess the collinearity of variables in the logistic regression. Finally, the effectiveness of the model was evaluated using the area under the curve (AUC), calibration curve, and decision curve analysis (DCA) curve.

## 3. Results

### 3.1. Multiple imputation

To avoid data leakage between the MCE and non-MCE groups, multiple imputations were performed for each group. The results and details are shown in Supplementary Figures 1, 2.

### 3.2. Demographic characteristics and clinical information

A total of 312 patients with acute LHI involving the anterior circulation were recruited in this study, among which 38.5% of the patients were categorized into the MCE group (*n* = 120). The baseline characteristics and clinical information of the study patients are shown in Table 1.

**Table 1:** Demographic characteristics and clinical information of the study patients (*n* = 312).

**Characteristics**	**All (*n* = 312)**	**Non-MCE group (*n* = 192)**	**MCE group (*n* = 120)**	***p*-value**
Age (years)	75.82 ± 12.18	75.21 ± 12.08	76.79 ± 12.32	0.265
Admission temperature (°C)	36.61 ± 0.61	36.53 ± 0.54	36.74 ± 0.69	0.002
Admission SBP (mmHg)	149.53 ± 23.18	149.60 ± 22.14	149.42 ± 24.85	0.945
Admission DBP (mmHg)	82.80 ± 14.75	83.42 ± 14.40	81.81 ± 15.32	0.35
GCS score	9.84 ± 3.56	11.17 ± 3.20	7.71 ± 3.04	<0.001
NIHSS score	16.87 ± 6.46	14.27 ± 5.71	21.03 ± 5.32	<0.001
ASPECTS	5.30 ± 3.08	6.59 ± 2.45	3.23 ± 2.85	<0.001
CS	1.53 ± 1.09	1.91 ± 0.98	0.92 ± 0.97	<0.001
WBC count	10.63 ± 3.60	9.74 ± 3.24	12.06 ± 3.69	<0.001
Neutrophil count	9.14 ± 7.26	8.12 ± 6.43	10.79 ± 8.18	0.001
Lymphocyte count	1.23 ± 0.64	1.32 ± 0.62	1.08 ± 0.65	0.001
NLR	10.63 ± 13.03	8.34 ± 9.10	14.30 ± 17.00	<0.001
RDW	13.54 ± 1.84	13.44 ± 1.80	13.70 ± 1.91	0.232
Total cholesterol level (mmol/L)	4.38 ± 1.45	4.31 ± 1.20	4.50 ± 1.77	0.247
Triglyceride level (mmol/L)	1.29 ± 0.84	1.32 ± 0.70	1.24 ± 1.03	0.442
HDL level	1.34 ± 1.94	1.36 ± 2.45	1.31 ± 0.38	0.817
LDL level	2.47 ± 0.87	2.49 ± 0.85	2.44 ± 0.89	0.605
HCY level	16.38 ± 8.77	16.80 ± 9.64	15.70 ± 7.14	0.282
Blood sugar level	8.37 ± 6.42	8.14 ± 7.55	8.73 ± 3.99	0.434
Blood creatinine level	87.63 ± 37.77	83.68 ± 26.35	93.96 ± 50.49	0.019
Urea level	6.57 ± 3.38	6.36 ± 3.13	6.90 ± 3.73	0.171
D-dimer level	3.68 ± 6.55	2.72 ± 5.04	5.21 ± 8.22	0.001
HbA1c level	6.73 ± 1.77	6.55 ± 1.46	7.00 ± 2.15	0.029
Fibrinogen level	3.97 ± 2.33	3.81 ± 1.41	4.21 ± 3.30	0.142
Admission WBC count	9.64 ± 3.84	9.77 ± 3.84	9.43 ± 3.86	0.447
Admission neutrophil count	7.57 ± 3.82	7.57 ± 3.79	7.56 ± 3.89	0.991
Admission lymphocyte count	1.50 ± 0.93	1.55 ± 0.91	1.41 ± 0.95	0.197
Admission NLR	7.54 ± 7.03	6.94 ± 6.41	8.51 ± 7.85	0.054
Admission monocyte count	0.54 ± 0.28	0.56 ± 0.29	0.50 ± 0.25	0.076
Admission PLT level	208.86 ± 95.10	223.46 ± 109.44	185.51 ± 59.34	0.001
Admission PLR	179.12 ± 110.19	179.97 ± 112.38	177.76 ± 107.06	0.864
APACHE II score	13.26 ± 5.59	11.28 ± 4.94	16.43 ± 5.10	<0.001
CRP level	42.89 ± 53.76	33.86 ± 40.97	57.32 ± 67.21	<0.001
Sex				
Female	157 (50.32%)	94 (48.96%)	63 (52.5%)	0.622
Male	155(49.68%)	98 (51.04%)	57 (47.5%)	
TOAST classification				
Large-artery atherosclerosis	170 (54.49%)	120 (62.5%)	50 (41.67%)	<0.001
Cardioembolism	138 (44.23%)	68 (35.42%)	70 (58.33%)	
Stroke of other determined cause	4 (1.28%)	4 (2.08%)	0 (0%)	
Consciousness disorders				
No	108 (34.62%)	91 (47.4%)	17 (14.17%)	<0.001
Yes	204 (76.92%)	101 (52.6%)	103 (85.83%)	
History of hypertension				
No	72 (23.08%)	35 (18.23%)	37 (30.83%)	0.015
Yes	240 (76.92%)	157 (81.77%)	83 (69.17%)	
History of diabetes mellitus				
No	199 (63.78%)	119 (61.98%)	80 (66.67%)	0.473
Yes	113 (36.22%)	73 (38.02%)	40 (33.33%)	
History of coronary heart disease				
No	261 (83.65%)	159 (82.81%)	102 (85%)	0.726
Yes	51 (16.35%)	33 (17.19%)	18 (15%)	
Atrial fibrillation				
No	176 (56.41%)	127 (66.15%)	49 (40.83%)	<0.001
Yes	136 (43.59%)	65 (33.85%)	71 (59.17%)	
Cardiac insufficiency				
No	223 (71.47%)	146 (76.04%)	77 (64.17%)	0.033
Yes	89 (28.53%)	46 (23.96%)	43 (35.83%)	
History of stroke				
No	223 (71.47%)	130 (67.71%)	93 (77.5%)	0.083
Yes	89 (28.53%)	62 (32.29%)	27 (22.5%)	
Smoking history				
No	255 (81.73%)	153 (79.69%)	102 (85%)	0.303
Yes	57 (18.27%)	39 (20.31%)	18 (15%)	
Drinking history				
No	269 (86.22%)	163 (84.9%)	106 (88.33%)	0.491
Yes	43 (13.78%)	29 (15.1%)	14 (11.67%)	
Pneumonia				
No	96 (30.77%)	66 (34.38%)	30 (25%)	0.105
Yes	216 (69.23%)	126 (65.62%)	90 (75%)	
UTI				
No	283 (90.71%)	172 (89.58%)	111 (92.5%)	0.507
Yes	29 (9.29%)	20 (10.42%)	9 (7.5%)	
Gastrointestinal bleeding				
No	295 (94.55%)	182 (94.79%)	113 (94.17%)	1
Yes	17 (5.45%)	10 (5.21%)	7 (5.83%)	
Hemorrhagic transformation				
No	235 (75.32%)	149 (77.6%)	86 (71.67%)	0.294
Yes	77 (24.68%)	43 (22.4%)	34 (28.33%)	
Seizure				
No	302 (96.79%)	185 (96.35%)	117 (97.5%)	0.819
Yes	10 (3.21%)	7 (3.65%)	3 (2.5%)	
Admission anisocoria				
No	262 (83.97%)	178 (92.71%)	84 (70%)	<0.001
Yes	50 (16.03%)	14 (7.29%)	36 (30%)	
Admission gaze deviation				
No	159 (50.96%)	115 (59.9%)	44 (36.67%)	<0.001
Yes	153 (49.04%)	77 (40.1%)	76 (63.33%)	
Lesion side				
Left	148 (47.44%)	93 (48.44%)	55 (45.83%)	0.74
Right	164 (52.56%)	99 (51.56%)	65 (54.17%)	
Non-ischemic territory				
Yes	193 (61.86%)	135 (70.31%)	58 (48.33%)	<0.001
No	119 (38.14%)	57 (29.69%)	62 (51.67%)	

SBP, systolic blood pressure; DBP, diastolic blood pressure; GCS, Glasgow Coma Scale; NIHSS, National Institutes of Health Stroke Scale; ASPECTS, Alberta Stroke Program Early Computed Tomography Score; CS, collateral score; WBC, white blood cell; NLR, neutrophil-to-lymphocyte ratio; RDW, red blood cell distribution width; HDL, high-density lipoprotein cholesterol; LDL, low-density lipoprotein cholesterol; HCY, homocysteine; FIB, fibrinogen; PLT, platelet; PLR, platelet-to-lymphocyte ratio; APACHE II, acute physiology and chronic health evaluation; CRP, C-reactive protein; UTI, urinary tract infection.

### 3.3. Dataset division

Following the process of random grouping, no statistically significant disparities were observed between the training and validation groups in relation to the various indicators. Additional details are provided in Supplementary Table 2.

### 3.4. LASSO regression and variable screening

**Figure 1:**
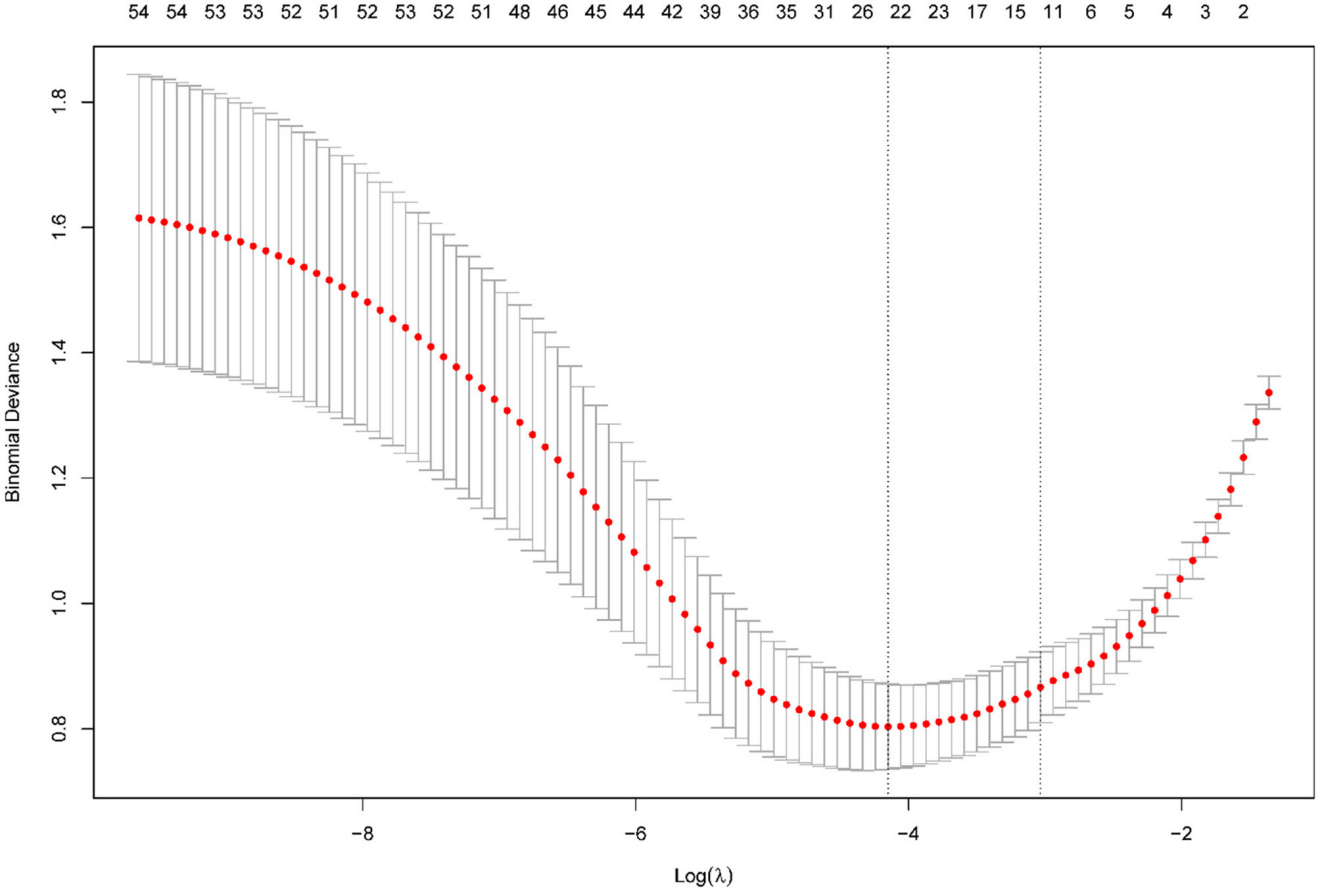
Results of the LASSO regression. Tuning parameter (lambda) selection in the LASSO model using 10-fold cross-validation via minimum criteria.

The LASSO regression analysis was performed with a minimum lambda value of 0.016 and one standard error (1SE) lambda value of 0.048. Figure 1 shows the regression results, wherein the vertical dashed line represents the 1SE lambda value selected by the algorithm using cross-validation.

**Figure 2:**
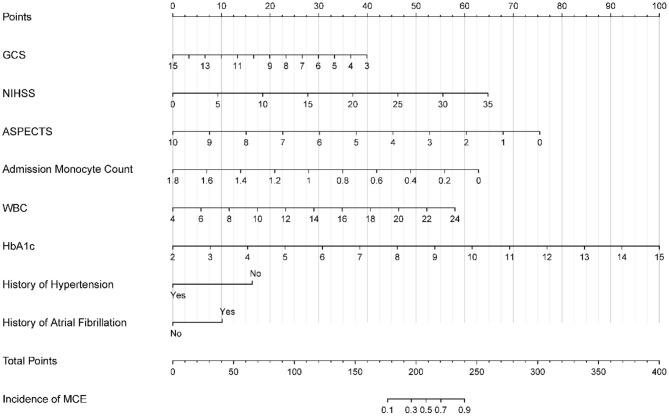
Nomogram for predicting MCE in patients with LHI. MCE, malignant cerebral edema; LHI, large hemisphere infarction.

As Table 2 shown, LASSO regression revealed the presence of 11 variables with non-zero coefficients (Figure 2), including pupil, Glasgow Coma Scale (GCS) score, acute physiology and chronic health evaluation (APACHE) II score, NIHSS score, ASPECTS, CS, admission monocyte count, white blood cell (WBC) count, HbA1c level, history of hypertension, and history of atrial fibrillation.

**Table 2:** LASSO regression results with coefficients of screened variables.

**Variables**	**LASSO coefficients**
Pupil	0.18824662
GCS score	−0.04460114
APACHE II score	0.0494358
NIHSS score	0.07749926
ASPECTS	−0.23724228
CS	−0.15449704
Admission monocyte count	−0.17134236
WBC count	0.01741388
HbA1c level	0.04377928
History of hypertension	−0.16209085
History of atrial fibrillation	0.05539656

GCS, Glasgow Coma Scale; APACHE II, acute physiology and chronic health evaluation; NIHSS, National Institutes of Health Stroke Scale; ASPECTS, Alberta Stroke Program Early Computed Tomography Score; CS, collateral score; WBC, white blood cell.

### 3.5. Logistic regression

Following the LASSO regression analysis, logistic regression was performed on the identified 11 non-zero coefficients. Subsequently, a backward stepwise regression was performed to reduce the AIC from 163.89 to 160.7, resulting in a final logistic regression model comprising eight variables, as shown in Table 3. Finally, the multicollinearity among the eight variables was tested using the variance inflation factor (also presented in Table 3), suggesting no significant collinearity.

**Table 3:** Univariate and multivariate logistic regression of the eight predictors.

**Variables**	**Univariate analysis**			**Multivariate analysis**			**VIF**
	**OR**	**95% CI**	***p*** **-value**	**OR**	**95% CI**	***p*** **-value**	
GCS score	0.68	0.60–0.75	<0.001	0.79	0.67–0.94	0.007	2.032
NIHSS score	1.23	1.16–1.32	<0.001	1.14	1.04–1.25	0.006	2.042
ASPECTS	0.66	0.58–0.74	<0.001	0.59	0.48–0.70	<0.001	1.153
Admission monocyte count	0.39	0.13–1.04	0.067	0.09	0.02–0.43	0.004	1.041
WBC count	1.22	1.13–1.33	<0.001	1.22	1.08–1.40	0.002	1.244
HbA1c level	1.19	1.01–1.40	0.040	1.71	1.28–2.35	<0.001	1.030
History of hypertension							1.019
No	1.00			1.00			
Yes	0.63	0.33–1.19	0.152	0.32	0.11–0.86	0.027	
History of atrial fibrillation							1.074
No	1.00			1.00			
Yes	2.35	1.35–4.11	0.003	2.03	0.85–4.96	0.114	

GCS, Glasgow Coma Scale; NIHSS, National Institutes of Health Stroke Scale; ASPECTS, Alberta Stroke Program Early Computed Tomography Score; WBC, white blood cell; OR, odds ratio; CI, confidence interval; VIF, variance inflation factor.

**Table 4:**
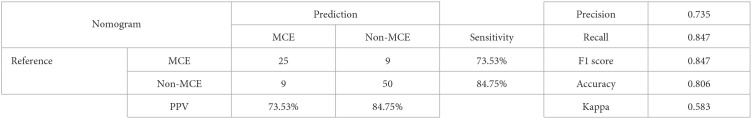
Confusion matrix from the nomogram.

### 3.6. Nomogram development and validation

A nomogram was developed based on the logistic regression results obtained from the training set and incorporated the eight identified factors (Figure 2). Each factor was assigned a score based on its corresponding scale, and the sum of the scores was used to determine the total points. By referencing the total points and the incidence rate shown in the “incidence of MCE” below, the risk of MCE occurrence in individuals with LHI could be evaluated.

**Figure 3:**
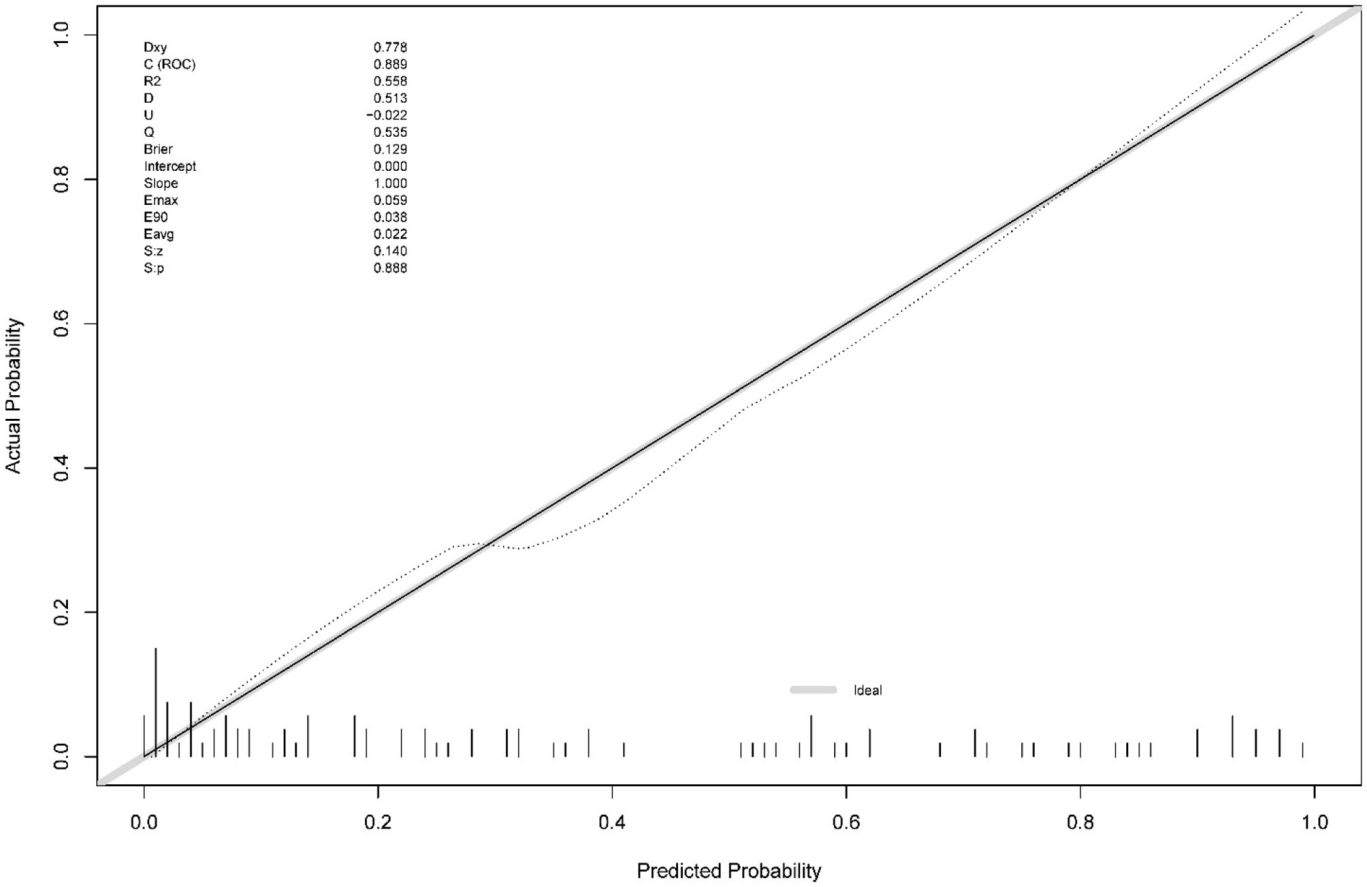
Calibration curve of the nomogram in the validation set.

The calibration curve (Figure 3) demonstrated a close match between the predicted probabilities by the logistic regression model and the observed incidence of MCE, indicating a good model fit. Therefore, the logistic regression model effectively predicted the occurrence of MCE in individuals with LHI.^1^,^2^,^3^

**Figure 4:**
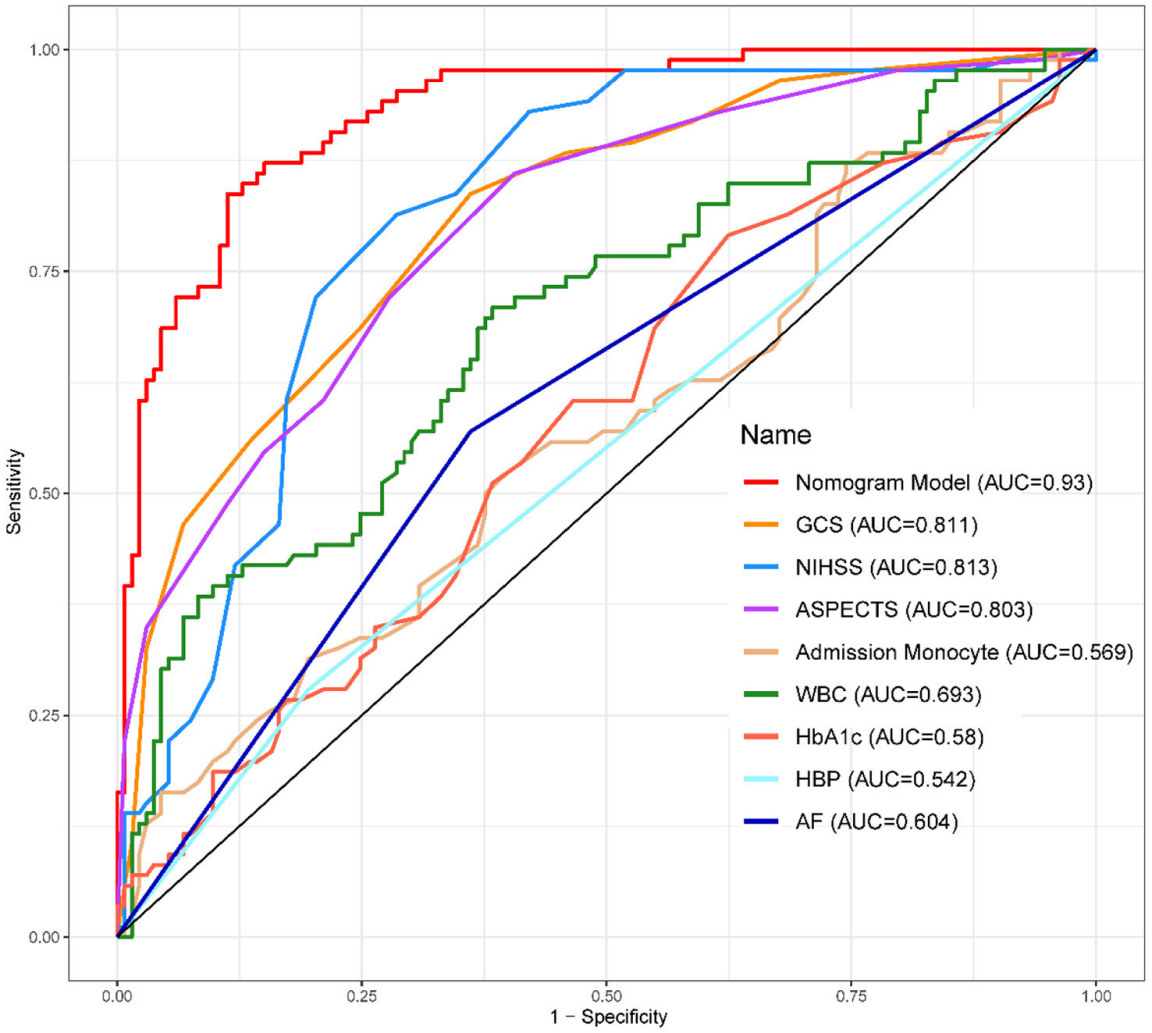
ROC curve of the training set. AUC, area under the curve.

**Figure 5:**
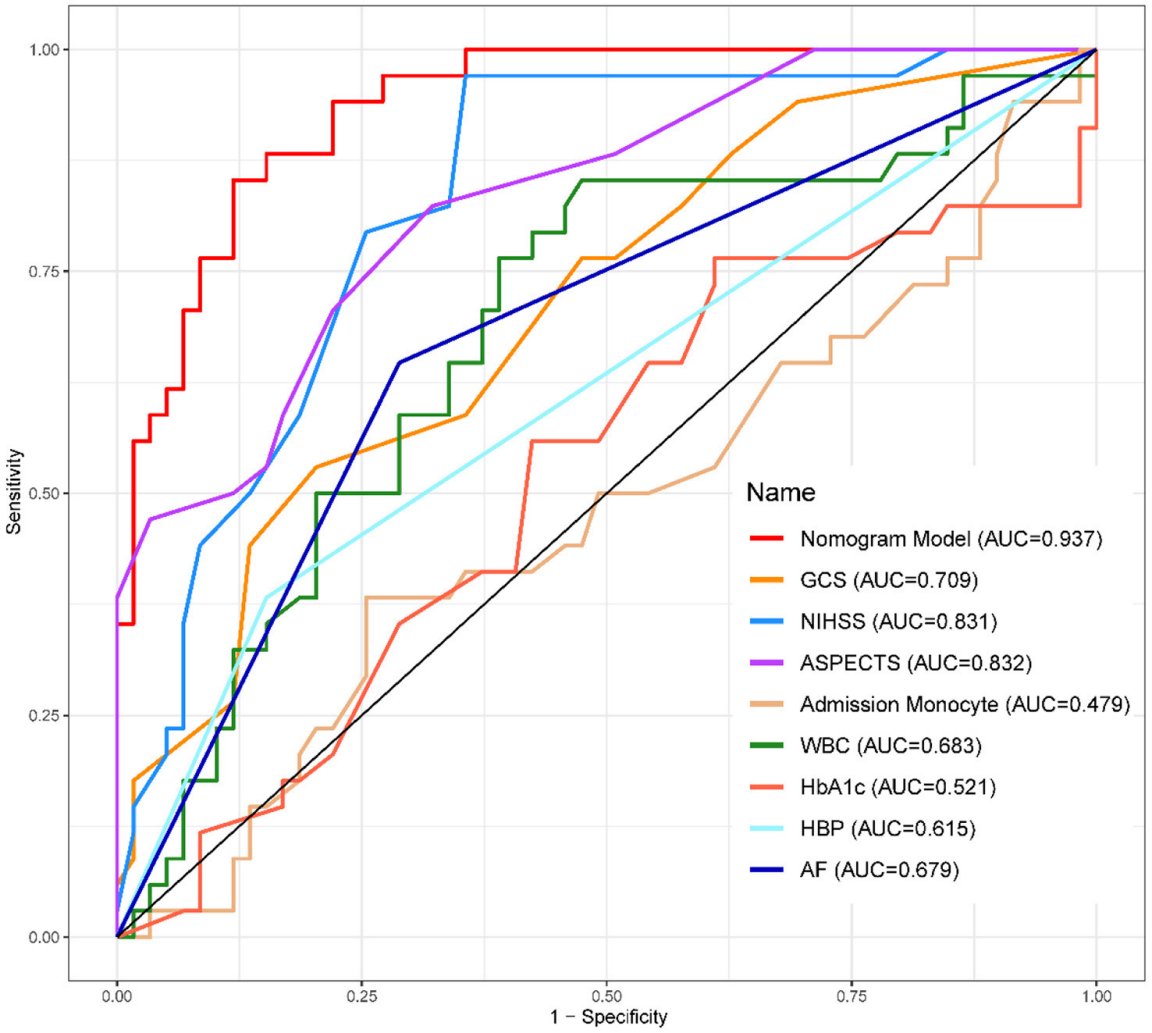
ROC curve of the validation set. AUC, area under the curve.

### 3.7. Clinical practice

**Figure 6:**
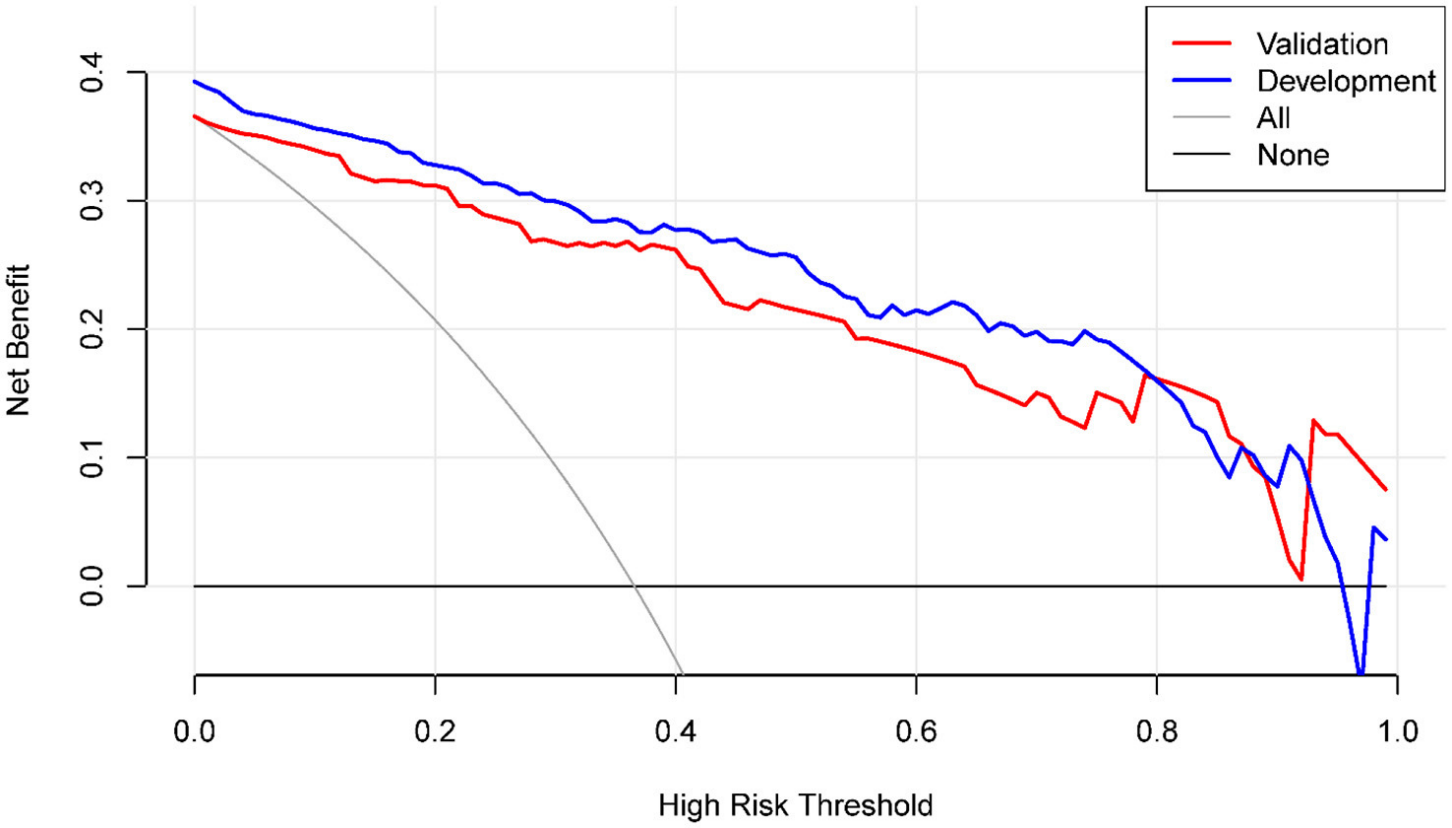
Decision curve analysis of the nomogram model.

The DCA curve (Figure 6) was used to evaluate the clinical utility of the nomogram. This analysis method considers the sensitivity and specificity of the model, as well as the costs and benefits of clinical decisions for assessing the net benefit of the model at different threshold probabilities. The DCA curve demonstrated that the nomogram had a high net benefit across the range of threshold probabilities, indicating good clinical utility. Therefore, our nomogram model has the potential to assist clinicians in making informed decisions concerning MCE risk in individuals with LHI.

**Figure 7:**
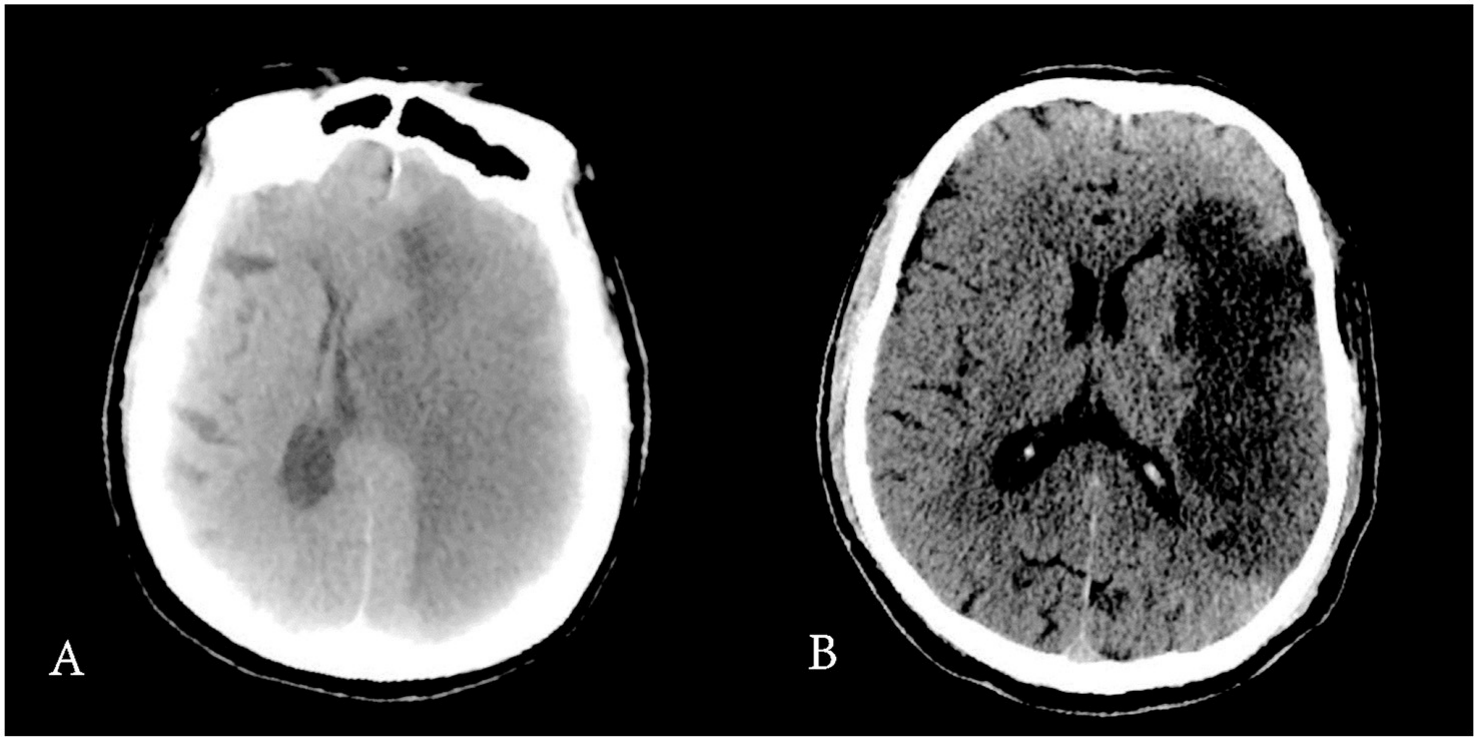
CT scan of MCE and non-MCE. **(A)** Refers to a MCE patient, **(B)** refers to a non-MCE patient.

## 4. Discussion

Among the 312 patients with LHI included in this study, 120 developed MCE, and all of them did not receive vascular recanalization treatment. We identified eight independent predictors of MCE, including GCS score, NIHSS score, ASPECTS, monocyte count, WBC count, HbA1c level, history of hypertension, and history of atrial fibrillation. Our study incorporated the serological and radiography indicators and clinical risk factors on admission into an easy-to-use nomogram to facilitate the individualized preoperative prediction of MCE. Although many of the screened predictors were consistent with those in other studies, some predictors found to have significant associations in certain previous studies were not independent predictors in our final model. The variation in results could be attributed to the inclusion criteria differences.

Compared to most studies, our study did not include patients who had received reperfusion therapy because rapid tissue reperfusion is recommended for treating acute ischemic stroke and successful reperfusion can limit the final infarct size and resultant edema. Moreover, we used the signs of herniation and brain swelling with a midline shift ≥5 mm to define MCE, regardless of whether DHC was performed.

NIHSS and GCS scores are highly predictive of long-term functional outcomes and have been widely used in clinical stroke trials and clinical practice, while the GCS score is used to assess consciousness levels for the neurological screening of patients during acute hospitalization. Additionally, a high NIHSS score is generally accepted in predicting MCE outcomes. Moreover, studies show that the NIHSS score can be used to predict MCE and be combined with other predictors to determine MCE risk (9).

Non-contrast CT, which is among the most commonly used primary imaging techniques at stroke centers for the initial evaluation of patients with acute ischemic stroke, has the advantages of being simple, easy to operate, and not time-consuming. ASPECTS is a simple quantitative tool to determine ischemic changes in the supratentorial MCA supply area (10 regions). This instrument is a surrogate marker of lesion size, with lower scores indicating larger lesion sizes and scores <6 predictive of LHI (3). Furthermore, the risk of a malignant course can be estimated by ASPECTS, where a cutoff score of 7 indicates progression to malignant infarction, with 50% sensitivity and 86% specificity (10). Moreover, the earlier the CT examination, the higher the specificity and the lower the sensitivity. ASPECTS has also been demonstrated to be a reliable and reproducible tool for quantifying the extent of parenchymal tissue damage after LHI (11). Diffusion-weighted imaging (DWI) can identify cerebral lesion volume within 6 h of symptom onset, while positron emission tomography and single-photon emission computed tomography could help determine tissue viability thresholds and elucidate the pathophysiological changes leading to space-occupying infarction. However, these indicators are not easily obtained because many centers limit their routine use during the acute stroke phase. However, evaluating ASPECTS on admission CT may compromise model sensitivity because patients arrive at different times after onset, and radiologic factors are usually visible in the later stage of stroke. Radiologic factors usually progress faster in patients who may ultimately develop fatal edema than in those who may not.

Circulating serum markers associated with the deterioration of BBB integrity have also been examined. Peripheral immune cells that are activated after stroke are most likely associated with BBB breakdown, cytokine release (12), and immune cell infiltration, which in turn influence the fate of the ischemic brain tissue. Furthermore, an increase in endothelial disruption has been linked with heightened inflammation, which results in elevated peripheral WBC count and immune cell infiltration. Moreover, systemic inflammation has been suggested to not merely reflect stroke severity but also play an essential role in stroke pathophysiology, stroke complications, and patient outcomes (13). The earliest inflammatory reaction observed 4–6 h after ischemia is leukocyte margination in the brain capillaries from the infarct zone and in the meninges (14). Systemic WBC count is a readily available blood test routinely conducted in all patients admitted with stroke.

Monocyte chemoattractant protein is a potent monocyte chemoattractant associated with good pretreatment collateral status (15), which could explain the positive relationship between monocyte count and MCE in our study. Increased circulating monocytes and macrophage infiltration due to BBB breakdown may help repair the injured brain and improve clinical outcomes (15). The recruited monocytes may also contribute to the resolution of ischemic inflammation through the phagocytosis of apoptotic neutrophils and cellular debris (12). However, the NLR did not demonstrate statistical significance in our study. This difference in result can be partially ascribed to the inclusion of only a few patients with hemorrhagic transformation and the exclusion of patients who underwent reperfusion therapy, which usually leads to a significant increase in the NLR (16).

Many studies have determined that hyperglycemia is associated with worsened edema in patients with cerebral ischemia (17–19), with collateral status as a mediator of the mechanism. In contrast to other studies (20), our study indicated that a history of hypertension was a protective factor, whereas our finding of atrial fibrillation history as a hazardous factor was in line with previous research outcomes. This finding may also be related to collateral circulation. Similarly, our results supported the prior reports of increased frequency of hypertension in patients with large-artery atherosclerosis (LAA) and higher occurrence of atrial fibrillation in patients with cardioembolism (CE). Moreover, one study demonstrated that compared with CE, LAA was associated with different perfusion-weighted imaging–DWI and MR angiography–DWI mismatch profiles, which were likely due to better collaterals (21).

The good collateral flow in LAA may be explained by the occlusion speed. In patients with CE and cryptogenic embolism, arterial occlusion occurs abruptly, resulting in insufficient time for developing collaterals. Correspondingly, atrial fibrillation is associated with greater volumes of more severe baseline hypoperfusion, causing greater infarct growth, a higher likelihood of severe hemorrhagic transformation, and poorer stroke outcomes (22). In contrast, complete arterial occlusion occurs over a longer time in LAA, allowing the development of adequate collateral flow before the stroke onset. Atrial fibrillation is also associated with an impaired cardiac output due to inadequate compensation by cerebral autoregulation (23).

A combined approach using some or all the indicators mentioned above may provide sufficient robustness for MCE prediction. Previous studies have identified several risk factors for MCE development (9–13, 24). However, these findings remain unclear due to the varied research populations and methods used. Although a few prior studies have addressed this issue using relatively small groups of carefully selected patients with stroke, these results may not apply to most cases of large strokes encountered in clinical practice. The most common limitation of the previous studies was the overfitting problem. Thus, the method to avoid overfitting is important for accurate prediction in clinical applications. Due to the reality that most patients experience delays in seeking emergency medical care, often surpassing the 24-h time frame, it is no longer feasible to provide vascular recanalization treatment in instances where LHI has progressed. Consequently, all patients involved in this study did not receive this form of treatment.

The strength of our predictive model is that it could determine MCE risk using the parameters available on admission. Furthermore, we clarified that this prediction tool was reliable when used as early as within 6 h up to 48 h after stroke among patients with severe stroke. Moreover, we used this model to assign concrete scores to important variables that influence important and clinically relevant outcomes.

It is worth mentioning that the differential diagnosis of malignant large hemispheric infarction also involves hematological disorders, which are often overlooked as a potential cause of ischemic stroke. Additionally, ischemic stroke can be a manifestation of hematological disorders, and hematological disorders are the most common etiology for cryptogenic ischemic strokes (25).

This study has certain limitations. First, this was a single-center retrospective cohort study with small sample size. Therefore, further external verification using a large prospective multi-center cohort is required. Second, we did not collect data on outcomes other than mortality, and severe dependency after stroke could have affected future treatment decisions. Finally, this study did not provide the time of edema occurrence. Considering that edema evolves over time, future studies incorporating the temporal dynamics of the prediction factors will be needed to elucidate the effect of changes in these factors on edema prediction as the ischemic stroke progresses.

## 5. Conclusion

Our study successfully established a prediction model using GCS score, NIHSS score, ASPECTS, monocyte count, WBC count, HbA1c level, history of hypertension, and history of atrial fibrillation as early indicators of MCE in patients with LHI who did not receive vascular recanalization treatment. Furthermore, these predictors may be more generalizable than those in prior studies because they were derived from a broad population on admission. Finally, our nomogram can help neurologists make clinical decisions and discuss prognosis with patient families, as well as provides guiding information for transfer to tertiary care centers, family discussions, surgery, and future research studies. Furthermore, we hope that future research can explore and expand upon pharmacological interventions for the acute phase of MCE.

## Data availability statement

Original data can be accessed from corresponding author under reasonable request.

## Ethics statement

The studies involving human participants were reviewed and approved by the Ethics Review Board of the Third Affiliated Hospital of Soochow University (2023-S-080). Written informed consent for participation was not required for this study in accordance with the national legislation and the institutional requirements.

## Author contributions

SS, ML, JY, and JW developed the research idea and designed the study. WX, XM, GX, YW, and WH performed the statistical analysis and wrote the manuscript. All authors contributed to the article and approved the submitted version.

## Footnotes

^1^The ROC Curves of development (Figure 4) and validation group (Figure 5) indicate that the Nomogram model had a great discrimination in risk prediction.

^2^Figure 7 showed the difference of CT scan in two patients.

^3^As Table 4 presented, the nomogram model had excellence performance and met the needs of clinical usage.
