# Supplementary material for: Development and validation of a nomogram for the risk prediction of malignant cerebral edema after acute large hemispheric infarction involving the anterior circulation
Source: Front Neurol. 2023 Sep 14;14:1221879. doi: 10.3389/fneur.2023.1221879 (PMC10538642; doi:10.3389/fneur.2023.1221879)
Supplement: Supplementary file 1 [file Data_Sheet_1.docx]

Supplementary Material

Development and Validation of a Nomogram for Predicting the risk of Malignant Cerebral Edema after Acute Anterior Circulating Large Hemisphere Infarction

# Data Process

The variables were subjected to processing, and the resulting table (**Supplementary Table 1.**) depicts the missing values and their corresponding proportions in the two groups.

Supplementary Table 1. Processed data and missing proportion in MCE & Normal Groups.

| **Variable Names** | **Classification** | **Missing in MCE Group n(%)** | **Missing in non-MCE Group n(%)** |
| --- | --- | --- | --- |
| Gender | Categoric | 0 (0%) | 0 (0%) |
| Age (years) | Numeric | 0 (0%) | 0 (0%) |
| TOAST | Numeric | 1 (0.8%) | 0 (0%) |
| Admission Temperature (°C) | Numeric | 0 (0%) | 0 (0%) |
| Admission SBP (mmHg) | Numeric | 0 (0%) | 0 (0%) |
| Admission DBP (mmHg) | Numeric | 0 (0%) | 0 (0%) |
| Consciousness | Categoric | 0 (0%) | 0 (0%) |
| Seizure | Categoric | 1 (0.8%) | 0 (0%) |
| Anisocoria | Categoric | 0 (0%) | 1 (0.5%) |
| Admission Gaze deviation | Categoric | 0 (0%) | 0 (0%) |
| GCS | Numeric | 0 (0%) | 1 (0.5%) |
| APACHE II | Numeric | 6 (5%) | 3 (1.6%) |
| NIHSS | Numeric | 0 (0%) | 1 (0.5%) |
| ASPECT | Numeric | 7 (5.8%) | 2 (1%) |
| CS | Numeric | 38 (31.7%) | 29 (15.1%) |
| Side | Categoric | 2 (1.7%) | 0 (0%) |
| Non-Ischemic Territory | Categoric | 3 (2.5%) | 0 (0%) |
| Infarct Volume | Numeric | 6 (5%) | 3 (1.6%) |
| WBC Emergency | Numeric | 3 (2.5%) | 3 (1.6%) |
| Neutrophil Count Emergency | Numeric | 3 (2.5%) | 3 (1.6%) |
| Lymphocyte Count Emergency | Numeric | 3 (2.5%) | 3 (1.6%) |
| NLR Emergency | Numeric | 3 (2.5%) | 3 (1.6%) |
| Monocyte Count Emergency | Numeric | 3 (2.5%) | 3 (1.6%) |
| Admission PLT level | Numeric | 3 (2.5%) | 3 (1.6%) |
| Admission PLR | Numeric | 4 (3.3%) | 3 (1.6%) |
| WBC | Numeric | 5 (4.2%) | 14 (7.3%) |
| Neutrophil Count | Numeric | 6 (5%) | 13 (6.8%) |
| Lymphocyte Count | Numeric | 6 (5%) | 13 (6.8%) |
| NLR | Numeric | 7 (5.8%) | 13 (6.8%) |
| CRP | Numeric | 14 (11.7%) | 26 (13.5%) |
| RDW | Numeric | 2 (1.7%) | 1 (0.5%) |
| Total Cholesterol | Numeric | 3 (2.5%) | 2 (1%) |
| Triglyceride | Numeric | 3 (2.5%) | 5 (2.6%) |
| HDL | Numeric | 3 (2.5%) | 3 (1.6%) |
| LDL | Numeric | 3 (2.5%) | 3 (1.6%) |
| HCY | Numeric | 9 (7.5%) | 11 (5.7%) |
| Blood Sugar | Numeric | 3 (2.5%) | 1 (0.5%) |
| Blood Creatinine | Numeric | 3 (2.5%) | 1 (0.5%) |
| Urea | Numeric | 3 (2.5%) | 1 (0.5%) |
| HbA1c | Numeric | 25 (20.8%) | 36 (18.8%) |
| D-Dimer | Numeric | 2 (1.7%) | 7 (3.6%) |
| Fib | Numeric | 3 (2.5%) | 5 (2.6%) |
| Pneumonia | Categoric | 0 (0%) | 0 (0%) |
| Ventilation | Categoric | 0 (0%) | 1 (0.5%) |
| UTI | Categoric | 0 (0%) | 0 (0%) |
| Gastrointestinal bleeding | Categoric | 0 (0%) | 0 (0%) |
| Hemorrhagic Transformation | Categoric | 0 (0%) | 0 (0%) |
| History of Hypertension | Categoric | 0 (0%) | 0 (0%) |
| History of Diabetes | Categoric | 0 (0%) | 0 (0%) |
| History of Atrial Fibrillation | Categoric | 0 (0%) | 0 (0%) |
| History of Coronary Heart Disease | Categoric | 1 (0.8%) | 0 (0%) |
| Cardiac Insufficiency | Categoric | 0 (0%) | 0 (0%) |
| History of Stroke | Categoric | 0 (0%) | 0 (0%) |
| History of Smoke | Categoric | 0 (0%) | 0 (0%) |
| History of Drink | Categoric | 0 (0%) | 1 (0.5%) |

Note: SBP, systolic blood pressure; DBP, diastolic blood pressure; GCS, Glasgow Coma Scale; NIHSS, National Institutes of Health Stroke Scale; ASPECTS, Alberta Stroke Program Early Computed Tomography Score; CS, collateral score; WBC, white blood cell; NLR, neutrophil-to-lymphocyte ratio; RDW, red blood cell distribution width; HDL, high-density lipoprotein cholesterol; LDL, low-density lipoprotein cholesterol; HCY, homocysteine; FIB, fibrinogen; PLT, platelet; PLR, platelet-to-lymphocyte ratio; APACHE II, acute physiology and chronic health evaluation; CRP, C-reactive protein; UTI, urinary tract infection.

# Dataset Division

Supplementary Table 2. Training & Validation Groups Characteristics.

| Characteristic | All (n=312) | Validation Group (n=93) | Training Group (n=219) | *p*-value |
| --- | --- | --- | --- | --- |
| Age (years) | 75.82±12.18 | 76.55±12.86 | 75.51±11.89 | 0.49 |
| Admission temperature (°C) | 36.61±0.61 | 36.60±0.57 | 36.62±0.63 | 0.82 |
| Admission SBP (mmHg) | 149.53±23.18 | 149.03±21.66 | 149.74±23.83 | 0.804 |
| Admission DBP (mmHg) | 82.80±14.75 | 82.45±13.35 | 82.95±15.34 | 0.787 |
| GCS | 9.84±3.56 | 10.32±3.53 | 9.63±3.55 | 0.116 |
| NIHSS | 16.86±6.46 | 16.30±6.31 | 17.10±6.52 | 0.318 |
| ASPECTS | 5.30±3.07 | 5.51±3.28 | 5.22±2.98 | 0.452 |
| CS | 1.53±1.09 | 1.56±1.08 | 1.51±1.09 | 0.724 |
| WBC count | 10.63±3.60 | 10.46±3.26 | 10.71±3.74 | 0.577 |
| Neutrophil count | 9.14±7.26 | 8.95±8.57 | 9.23±6.65 | 0.763 |
| Lymphocyte count | 1.23±0.64 | 1.23±0.65 | 1.23±0.64 | 0.987 |
| NLR | 10.57±13.02 | 9.77±10.93 | 10.91±13.85 | 0.558 |
| RDW | 13.54±1.84 | 13.77±2.12 | 13.44±1.71 | 0.148 |
| Total Cholesterol | 4.38±1.45 | 4.52±2.01 | 4.32±1.13 | 0.263 |
| Triglyceride | 1.29±0.84 | 1.23±0.54 | 1.31±0.94 | 0.41 |
| HDL | 1.34±1.94 | 1.23±0.54 | 1.31±0.94 | 0.41 |
| LDL | 2.47±0.88 | 2.46±0.88 | 2.47±0.88 | 0.928 |
| HCY | 16.40±8.49 | 17.74±10.18 | 15.84±7.62 | 0.07 |
| Blood Sugar | 8.40±6.42 | 7.80±3.38 | 8.65±7.33 | 0.288 |
| Blood Creatinine | 87.43±36.46 | 87.01±40.11 | 87.60±34.89 | 0.896 |
| Urea | 6.54±3.30 | 6.55±2.42 | 6.53±3.61 | 0.974 |
| D-Dimer | 3.66±6.51 | 3.71±5.39 | 3.64±6.94 | 0.937 |
| HbA1c | 6.70±1.66 | 6.77±1.58 | 6.67±1.70 | 0.643 |
| Fib | 3.97±2.33 | 4.08±2.48 | 3.93±2.26 | 0.605 |
| Admission WBC | 9.68±3.82 | 9.66±3.63 | 9.69±3.91 | 0.952 |
| Admission neutrophil count | 7.62±3.81 | 7.63±3.74 | 7.62±3.85 | 0.983 |
| Admission lymphocyte count | 1.49±0.93 | 1.43±0.88 | 1.51±0.95 | 0.48 |
| Admission NLR | 7.58±7.01 | 7.94±7.71 | 7.43±6.70 | 0.562 |
| Admission monocyte count | 0.54±0.28 | 0.53±0.28 | 0.55±0.28 | 0.536 |
| Admission PLT level | 209.60±94.76 | 205.20±90.16 | 211.46±96.79 | 0.595 |
| Admission PLR | 180.58±112.24 | 179.81±95.53 | 180.90±118.84 | 0.938 |
| APACHE II | 13.17±5.52 | 13.68±5.20 | 12.95±5.64 | 0.287 |
| CRP | 42.55±53.41 | 37.89±44.98 | 44.53±56.60 | 0.316 |
| Sex |  |  |  |  |
| Female | 157 (50.32%) | 51 (54.84%) | 106 (51.6%) | 0.359 |
| Male | 155(49.68%) | 42 (45.16%) | 113 (56.62%) |  |
| TOAST |  |  |  |  |
| Large Artery Atherosclerosis | 170 (54.49%) | 46 (49.46%) | 124 (56.62%) | 0.173 |
| Cardioembolic | 138 (44.23%) | 47 (50.54%) | 91 (41.55%) |  |
| Stroke of other determined cause | 4 (1.28%) | 0 (0%) | 4 (1.83%) |  |
| Consciousness disorders |  |  |  |  |
| No | 108 (34.62%) | 32 (34.41%) | 76 (34.70%) | 1 |
| Yes | 204 (76.92%) | 61 (65.59%) | 143 (65.30%) |  |
| History of Hypertension |  |  |  |  |
| No | 72 (23.08%) | 23 (24.73%) | 49 (22.37%) | 0.76 |
| Yes | 240 (76.92%) | 70 (75.27%) | 170 (77.63%) |  |
| History of Diabetes Mellitus |  |  |  |  |
| No | 199 (63.78%) | 63 (67.74%) | 136 (62.10%) | 0.412 |
| Yes | 113 (36.22%) | 30 (32.26%) | 83 (37.90%) |  |
| History of Coronary Heart Disease |  |  |  |  |
| No | 261 (83.65%) | 79 (84.95%) | 182 (83.11%) | 0.814 |
| Yes | 51 (16.35%) | 14 (15.05%) | 37 (16.89%) |  |
| Atrial Fibrillation |  |  |  |  |
| No | 176 (56.41%) | 45 (48.39%) | 131 (59.82%) | 0.082 |
| Yes | 136 (43.59%) | 48 (51.61%) | 88 (40.18%) |  |
| Cardiac Insufficiency |  |  |  |  |
| No | 223 (71.47%) | 64 (68.82%) | 159 (72.60%) | 0.589 |
| Yes | 89 (28.53%) | 29 (31.18%) | 60 (27.40%) |  |
| History of Stroke |  |  |  |  |
| No | 223 (71.47%) | 67 (72.04%) | 156 (71.23%) | 0.994 |
| Yes | 89 (28.53%) | 26 (27.96%) | 63 (28.77%) |  |
| Smoke |  |  |  |  |
| No | 255 (81.73%) | 82 (88.17%) | 187 (85.39%) | 0.636 |
| Yes | 57 (18.27%) | 11 (11.83%) | 32 (14.61%) |  |
| Drink |  |  |  |  |
| No | 269 (86.22%) | 82 (88.17%) | 187 (85.39%) | 0.636 |
| Yes | 43 (13.78%) | 11 (11.83%) | 32 (14.61%) |  |
| Pneumonia |  |  |  |  |
| No | 96 (30.77%) | 30 (32.26%) | 66 (30.14%) | 0.812 |
| Yes | 216 (69.23%) | 63 (67.74%) | 153 (69.86%) |  |
| UTI |  |  |  |  |
| No | 283 (90.71%) | 83 (89.25%) | 200 (91.32%) | 0.715 |
| Yes | 29 (9.29%) | 10 (10.75%) | 19 (8.68%) |  |
| Gastrointestinal bleeding |  |  |  |  |
| No | 295 (94.55%) | 90 (96.77%) | 205 (93.61%) | 0.393 |
| Yes | 17 (5.45%) | 3 (3.23%) | 14 (6.39%) |  |
| Hemorrhagic Transformation |  |  |  |  |
| No | 235 (75.32%) | 64 (68.82%) | 171 (78.08%) | 0.111 |
| Yes | 77 (24.68%) | 29 (31.18%) | 48 (21.92%) |  |
| Seizure |  |  |  |  |
| No | 302 (96.79%) | 91 (97.85%) | 211 (96.35%) | 0.735 |
| Yes | 10 (3.21%) | 2 (2.15%) | 8 (3.65%) |  |
| Admission anisocoria |  |  |  |  |
| No | 262 (83.97%) | 79 (84.95%) | 183 (83.56%) | 0.892 |
| Yes | 50 (16.03%) | 14 (15.05%) | 36 (16.44%) |  |
| Gaze |  |  |  |  |
| No | 159 (50.96%) | 46 (49.46%) | 113 (51.60%) | 0.825 |
| Yes | 153 (49.04%) | 47 (50.54%) | 106 (48.40%) |  |
| Lesion Side |  |  |  |  |
| Left | 148 (47.44%) | 43 (46.24%) | 105 (47.95%) | 0.879 |
| Right | 164 (52.56%) | 50 (53.76%) | 114 (52.05%) |  |
| Non-Ischemic Territory |  |  |  |  |
| Yes | 193 (61.86%) | 54 (58.06%) | 139 (63.47%) | 0.44 |
| No | 119 (38.14%) | 39 (41.94%) | 80 (36.53%) |  |

Note: SBP, systolic blood pressure; DBP, diastolic blood pressure; GCS, Glasgow Coma Scale; NIHSS, National Institutes of Health Stroke Scale; ASPECTS, Alberta Stroke Program Early Computed Tomography Score; CS, collateral score; WBC, white blood cell; NLR, neutrophil-to-lymphocyte ratio; RDW, red blood cell distribution width; HDL, high-density lipoprotein cholesterol; LDL, low-density lipoprotein cholesterol; HCY, homocysteine; FIB, fibrinogen; PLT, platelet; PLR, platelet-to-lymphocyte ratio; APACHE II, acute physiology and chronic health evaluation; CRP, C-reactive protein; UTI, urinary tract infection.

# Supplementary Figures

**
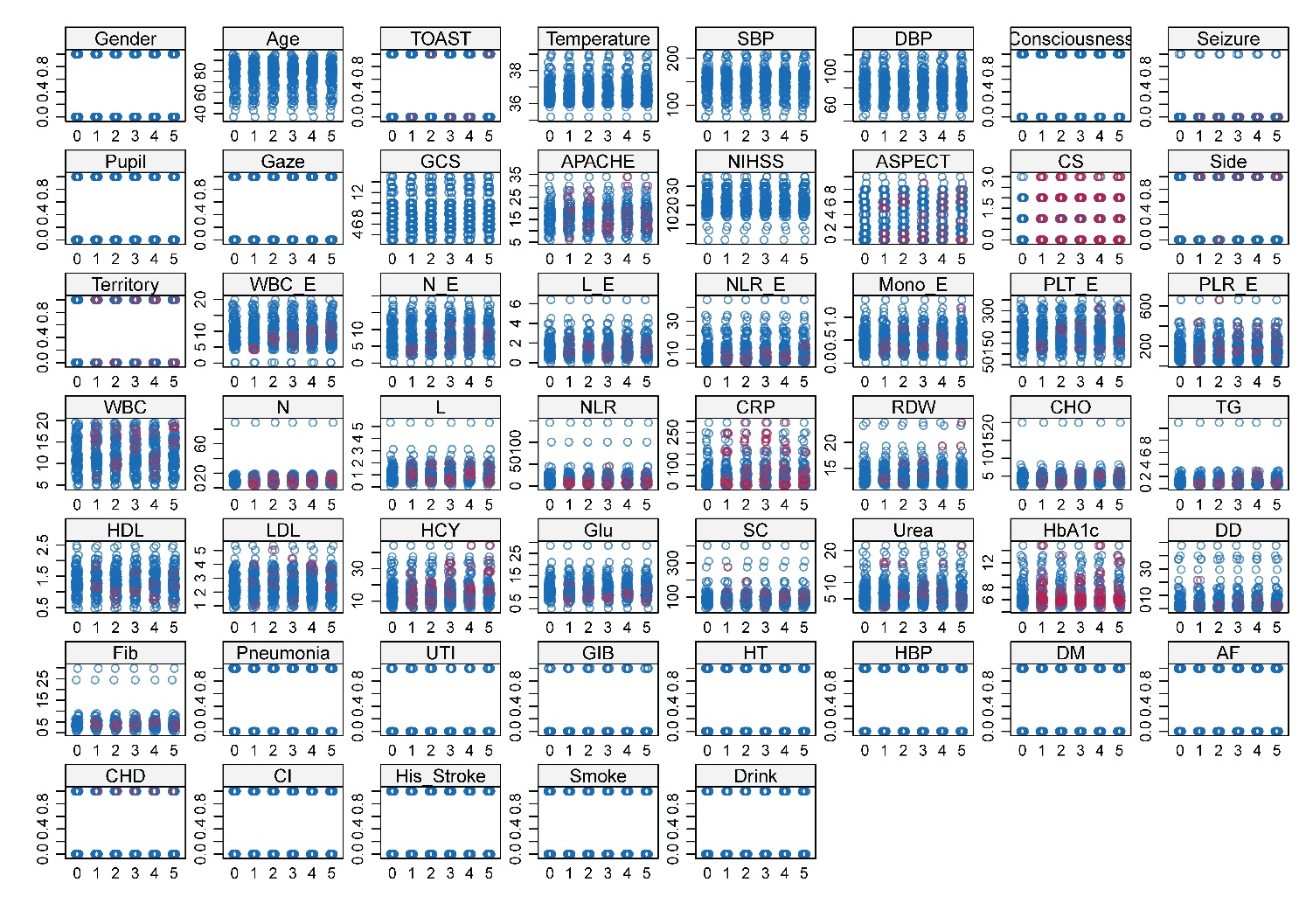
**

**Supplementary Figure 1.** **Multiple Imputation Result for MCE Group.** The interpolation output is represented by the red mark.

**
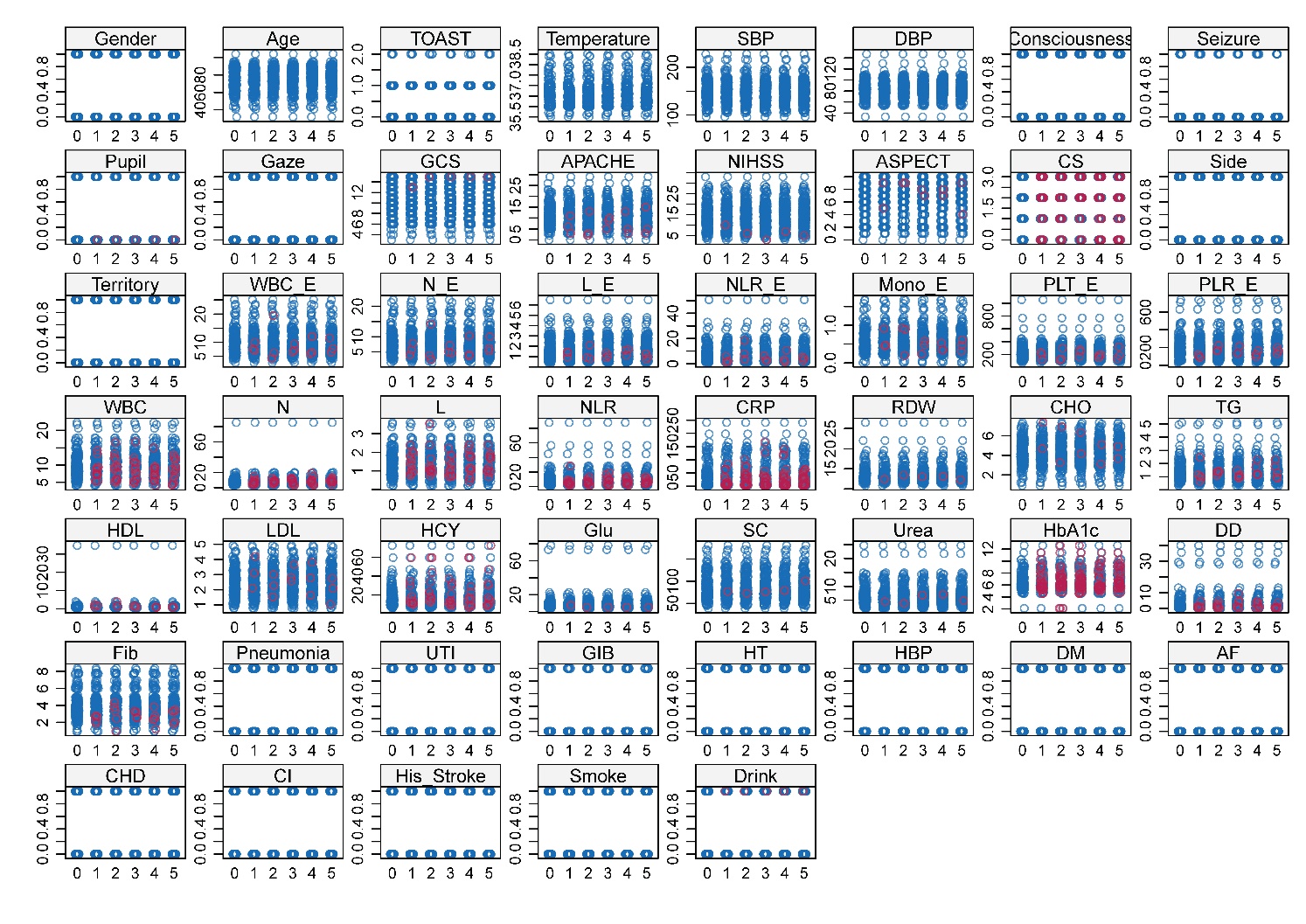
**

**Supplementary Figure 2. Multiple Imputation Result for non-MCE Group.** The interpolation output is represented by the red mark.
